# Partial protective effects of melatonin on developing brain in a rat model of chorioamnionitis

**DOI:** 10.1038/s41598-021-01746-w

**Published:** 2021-11-12

**Authors:** Geraldine Favrais, Elie Saliba, Léa Savary, Sylvie Bodard, Zuhal Gulhan, Pierre Gressens, Sylvie Chalon

**Affiliations:** 1 i-Brain Team- UMR INSERM U1253, UFR de Médecine, Université de Tours, Bâtiment Thérèse Planiol, 10 Bd Tonnellé, BP 3223, 37032 Tours Cedex 1, France; 2 Neonatology Unit, CHRU de Tours, Tours, France; 3 NeuroDiderot, Inserm, Université de Paris, Paris, France

**Keywords:** Developmental biology, Neuroscience

## Abstract

Melatonin has shown promising neuroprotective effects due to its anti-oxidant, anti-inflammatory and anti-apoptotic properties, making it a candidate drug for translation to humans in conditions that compromise the developing brain. Our study aimed to explore the impact of prenatal melatonin in an inflammatory/infectious context on GABAergic neurons and on oligodendrocytes (OLs), key cells involved in the encephalopathy of prematurity. An inflammatory/infectious agent (LPS, 300 μg/kg) was injected intraperitoneally (i.p.) to pregnant Wistar rats at gestational day 19 and 20. Melatonin (5 mg/kg) was injected i.p. following the same schedule. Immunostainings focusing on GABAergic neurons, OL lineage and myelination were performed on pup brain sections. Melatonin succeeded in preventing the LPS-induced decrease of GABAergic neurons within the retrospenial cortex, and sustainably promoted GABAergic neurons within the dentate gyrus in the inflammatory/infectious context. However, melatonin did not effectively prevent the LPS-induced alterations on OLs and myelination. Therefore, we demonstrated that melatonin partially prevented the deleterious effects of LPS according to the cell type. The timing of exposure related to the cell maturation stage is likely to be critical to achieve an efficient action of melatonin. Furthermore, it can be speculated that melatonin exerts a modest protective effect on extremely preterm infant brains.

## Introduction

Melatonin has shown several neuroprotective properties through the reduction of oxidative stress, proinflammatory response and apoptotic cell death ^1–6^. These effects of melatonin were associated with protection of the neonatal brain against hypoxic and inflammatory insults in several experimental models^2,3,7–9^. In parallel, melatonin showed a good safety profile^10,11^. From these encouraging results, melatonin appears to be one of the highest promising candidate drugs for translation to humans in conditions that compromise the developing brain^12,13^.

The encephalopathy of prematurity consists in destructive and developmental alterations of the cerebral white and grey matter due to one to several insults occurring from 23 to 32 weeks of gestation in humans (i.e., in rodents from the gestational day (GD) 19 to the postnatal day (PND) 5)^14^. GABAergic interneurons, oligodendrocyte precursor cells (OPCs) and pre-oligodendrocytes (Pre-OLs) are particularly vulnerable during this window of brain development^14^. A significant reduction in GABAergic neurons was observed in the white matter and in the cortical subplate of infants suffering from encephalopathy of prematurity^15^. In parallel, OPC and Pre-OL losses and axonal disruptions lead to focal necrotic lesions within the white matter^16^. Moreover, an arrest of oligodendrocyte (OL) lineage maturation at the Pre-OL stage induced a diffuse hypomyelination^17^.

In previous work, a rodent model was designed to explore the consequences of an infectious/inflammatory challenge performed during late pregnancy on the developing brain to mimic the chorioamnionitis context^18^. This infectious/inflammatory challenge consisted in intraperitoneal (i.p.) injections of a liposaccharide portion of Escherichia Coli membrane (LPS) to female rats at GD19 and at GD20. Transient hypomyelination was observed within the external capsule at PND7 in pup rats prenatally exposed to LPS, mimicking one hallmark of the encephalopathy of prematurity^18^.

Then, a strategy to prevent LPS effects on the neonatal brain was designed using melatonin in this model. Melatonin was injected within the pregnant-rat peritoneum at the same time as LPS at the dose of 5 mg/kg once a day at GD19 and at GD20. A first set of data has previously been published focusing on mechanisms that are implicated in neonatal brain injury pathophysiology^19^. This melatonin dosing regimen counteracted the sensitizing effect of LPS to a second excitotoxic insult consisting in ibotenate stereotaxic injection in the pup brain at PND4. Ibotenate-induced brain lesion size was reduced by 40% in pups exposed to LPS + Mel in comparison with LPS pups to meet a similar size to the control group^19^. Then, these results were encouraging for hoping that melatonin according to this schedule could sustainably prevent alterations induced by the LPS challenge on cell lineage development in this rodent model reproducing the encephalopathy of prematurity. The present study therefore aimed to explore the impact of melatonin on the GABAergic neurons and the OL lineage from PND1 to PND21 in control and inflammatory/infectious backgrounds.

## Results

### Prenatal LPS led to neonatal death and weight deficit at PND1 irrespective to the melatonin exposure

**Figure 1:**
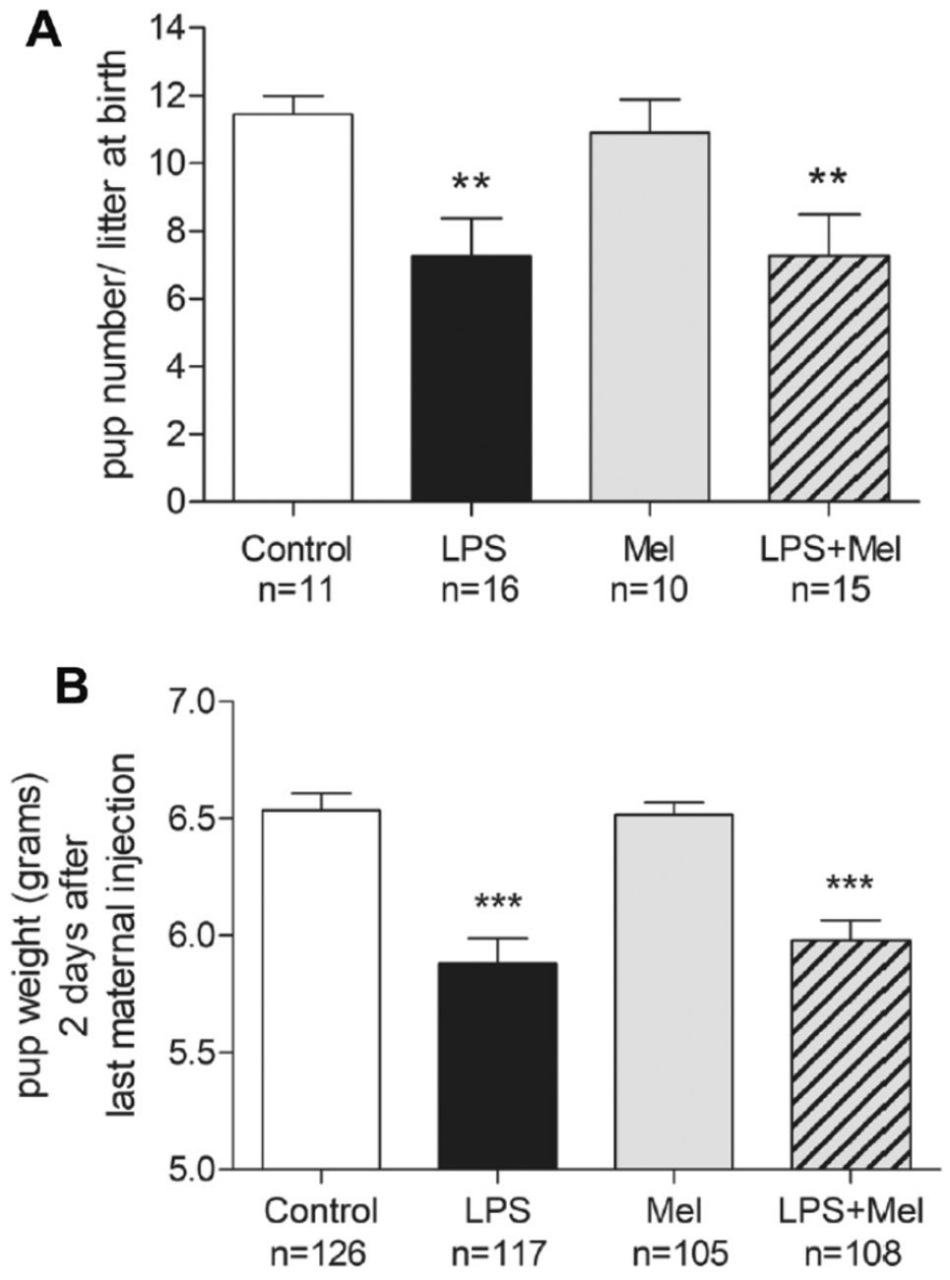
LPS injections to pregnant rats induced a reduction of pup number and pup weight at PND1 irrespective to melatonin exposure. (**A**) Graphic representation of the pup number per litter according to the experimental treatment. (**B**) Graphic representation of pup weight in grams at PND1, 2 days after the last maternal injection, according to the experimental treatment. White, black, grey and hatched grey bars represented the Control, the liposaccharide portion of the *E. coli* membrane (LPS), the melatonin (Mel) and the LPS + Mel groups, respectively. Results are expressed as mean ± standard deviation. Statistical analysis: One-Way-ANOVA test was performed associated with Bonferroni post-test, *p < 0.05 and ***p < 0.001 in comparison with the Control group.

The pup number per litter was significantly lower in the LPS group than in the control group at PND1 (median pup number per litter [range], control group: 12 [9–13] vs. LPS group: 8 [0–13], *p* = 0.007) (Fig. 1A). In parallel, the mean pup weight was reduced at PND1 after maternal exposure to LPS (median weight (in grams) [range] at PND1, control group: 6.5 [4.2–9.4] vs. LPS group: 6.1 [3.4–8.1], *p* < 0.0001) (Fig. 1B). Melatonin did not prevent the deleterious of LPS on these parameters.

### Systemic inflammation induced by LPS in PND1 pups was prevented by melatonin

**Figure 2:**
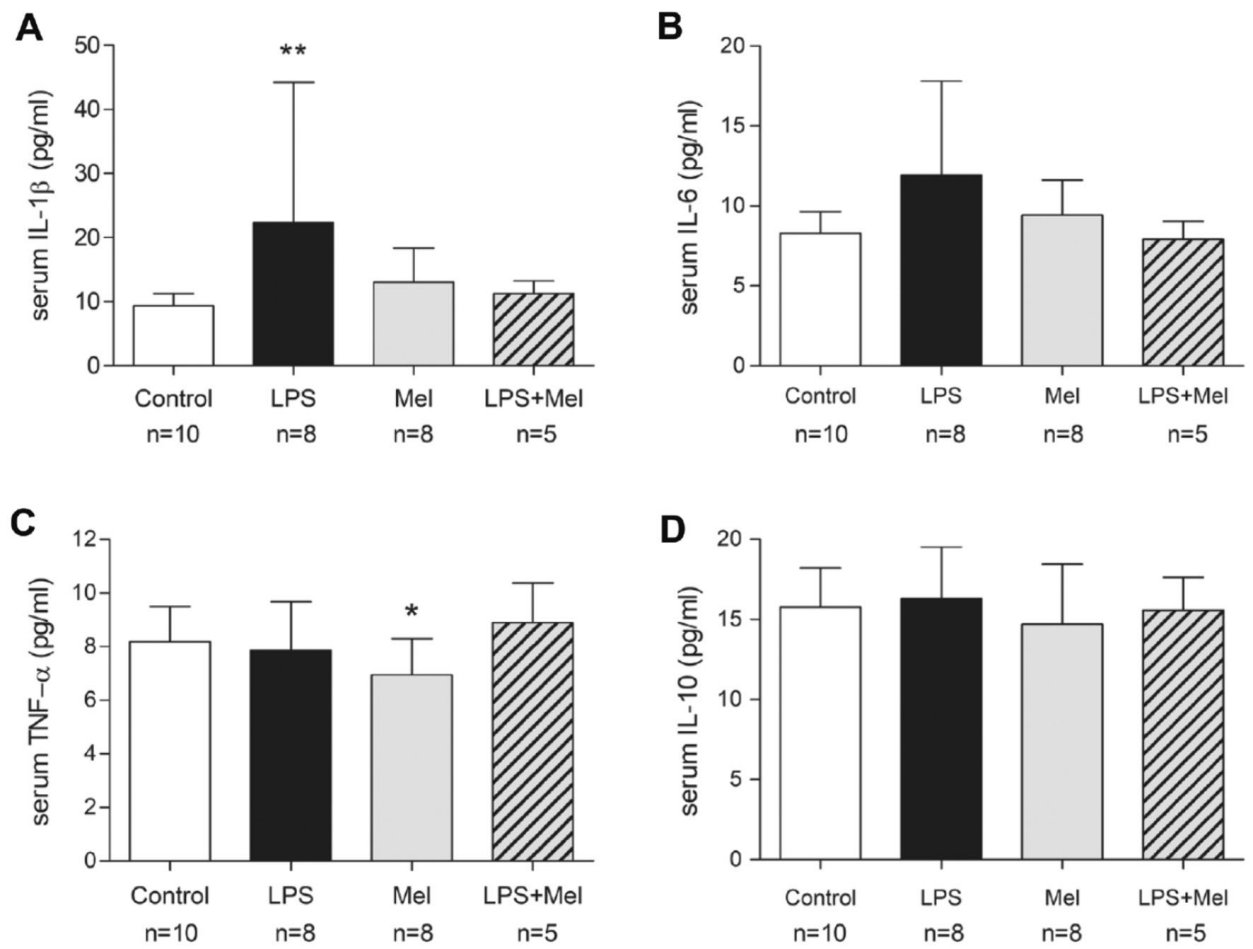
Melatonin prevented the LPS-induced inflammation in rat pup sera at PND1. Serum cytokine concentration in pg/ml were measured in pups at PND1 by multiplex assay. Each graph reports one cytokine measurement, i.e., IL-1β (**A**), IL-6 (**B**), TNF-α (**C**) and IL-10 (**D**). These measurements were performed according to our four experimental groups, i.e., the Control (Control), the liposaccharide portion of the *E. coli* membrane (LPS), the melatonin (Mel) and the LPS + Mel groups. Results are expressed as mean (bold line) ± standard deviation. Statistical analysis: One-Way-ANOVA test was performed associated with Bonferroni post-test, *p < 0.05 and **p < 0.01 in comparison with the Control group.

Acute inflammatory response is associated with the production of pro-inflammatory cytokines like IL-1β, Interleukin-6 (IL-6) and Tumor Necrosis Factor-α (TNF-α). In parallel, a trophic response is triggered including anti-inflammatory cytokines like Interleukin-10 (IL-10). In our model, higher concentration of IL-1β and a trend in higher IL-6 level were observed in the serum of PND1 pups exposed to LPS suggesting that LPS injections would induce systemic inflammation in fetal and newborn rats (*p* = 0.008 and *p* = 0.3, respectively) (Fig. 2A,B). Serum TNF-α and IL-10 was not altered by LPS in PND1 pups (Fig. 2C,D). Melatonin prevented this LPS-induced inflammatory response in PND1 pups. Furthermore, lower concentration of serum TNF-α was observed in the Mel group (*p* = 0.04) (Fig. 2C).

### Melatonin showed variable preventive effects on GABAergic neurons after LPS challenge depending on the grey matter region

**Figure 3:**
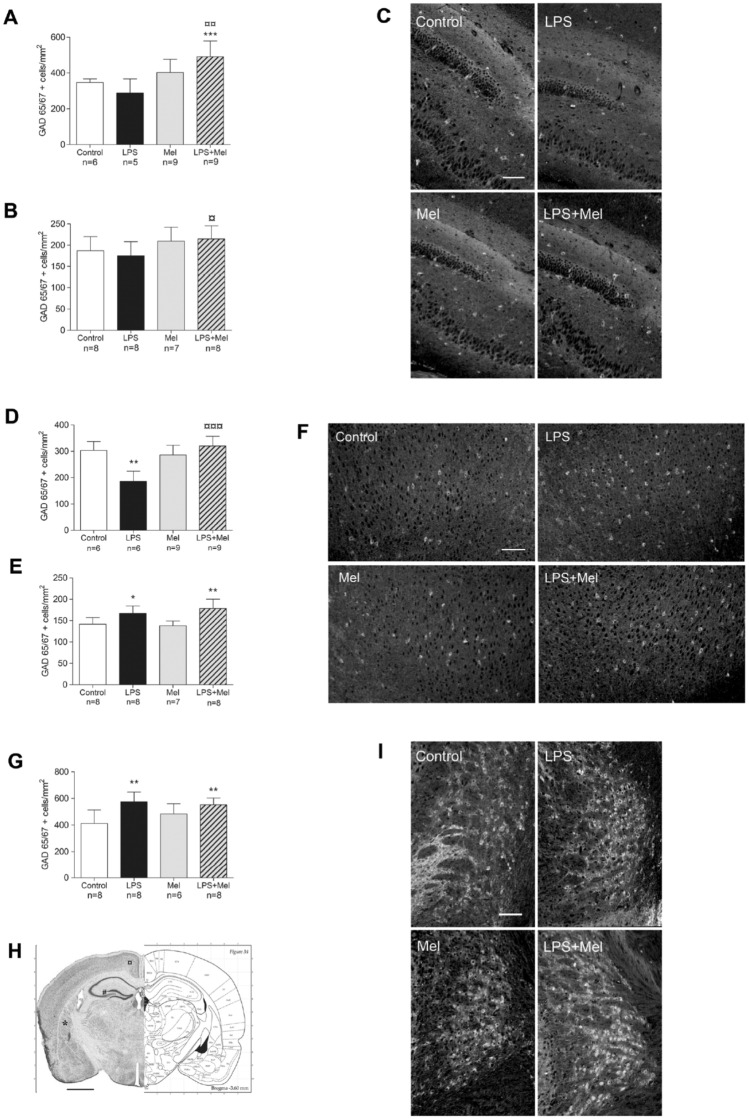
Melatonin demonstrated promoting effects on GABAergic neurons in the infectious/inflammatory context throughout the gray matter of the developing brain. The GAD 65/67 positive cells were counted at PND7 and at PND21 within the dentate gyrus hilus (**A,B**, respectively) and the retrospenial cortex (**D,E**, respectively), and are reported in the corresponding bar graphs. The GAD 65/67 positive cells were also counted at PND21 within the caudate-putamen area (**G**). Photomicrographs in grey scale of the GAD 65/67 immunostaining were performed on the PND21 pup brains from all experimental groups within the dentate gyrus (**C**), the retrospenial cortex (**F**) and the caudate-putamen area (**I**) (magnification 10, scale bar = 100 µm). Dentate gyrus, retrospenial cortex and caudate-putamen area were tagged with (#), (**¤**) and (*) on coronal rat brain sections extracted from the Paxinos Atlas, respectively (**H**)^50^. White, black, grey and hatched grey bars represented the Control, the liposaccharide portion of the *E.Coli* membrane (LPS), the melatonin (Mel) and the LPS + Mel groups, respectively. Results are expressed as the mean of positive cells/mm^2^ ± standard deviation. Statistical analysis: One-Way-ANOVA test was performed associated with Bonferroni post-test, *p < 0.05, **p < 0.01 and ***p < 0.001 in comparison with the Control group. ¤*p* < 0.05, ¤¤*p* < 0.01 and ¤¤¤*p* < 0.001 in comparison with the LPS group.

LPS did not show any significant effect on the GABAergic neuron population of the dentate gyrus at PND7 and at PND21 (*p* = 0.43 and *p* = 0.5 in comparison with the Control group, respectively). However, melatonin induced a significant and sustainable increase in GABAergic neurons in rats exposed to the LPS challenge (LPS + Mel group) (*p* = 0.004 at PND7 and *p* = 0.03 at PND21 in comparison with the LPS group, respectively) (Fig. 3A–C). This rise in GAD65/67-positive cells was only significant at PND7 in the LPS + Mel rats in comparison with the Control rats (*p* = 0.0008) (Fig. 3A).

A significant decrease in the GAD65/67-positive cells was observed within the retrospenial cortex in brains of PND7 rats exposed to LPS (*p* = 0.02 in comparison with the Control group) (Fig. 3D). Conversely, the GAD65/67-positive cells were significantly more numerous within the retrospenial cortex of PND21 brains in the LPS group (*p* = 0.01 in comparison with the Control group) (Fig. 3E,F). Melatonin prevented the LPS effect at PND7 and restored the GAD65/67 population within the retrospenial cortex (*p* = 0.0008 in comparison with the LPS group) (Fig. 3D). At PND21, melatonin in association with LPS demonstrated no specific effect and resulted in a similar increase in the GAD 65/67 population as in the LPS group (*p* = 0.002 in comparison with the Control group and *p* = 0.56 in comparison with the LPS group, respectively) (Fig. 3E,F). In the caudate-putamen region at PND21, the GABAergic neuron responses to LPS and/or to melatonin were similar to those observed in the retrospenial cortex at PND21 (*p* = 0.001 in comparison with the Control group and *p* = 0.46 in comparison with the LPS group) (Fig. 3G,I).

### Melatonin failed to prevent alterations of the oligodendrocyte lineage induced by the LPS challenge

**Figure 4:**
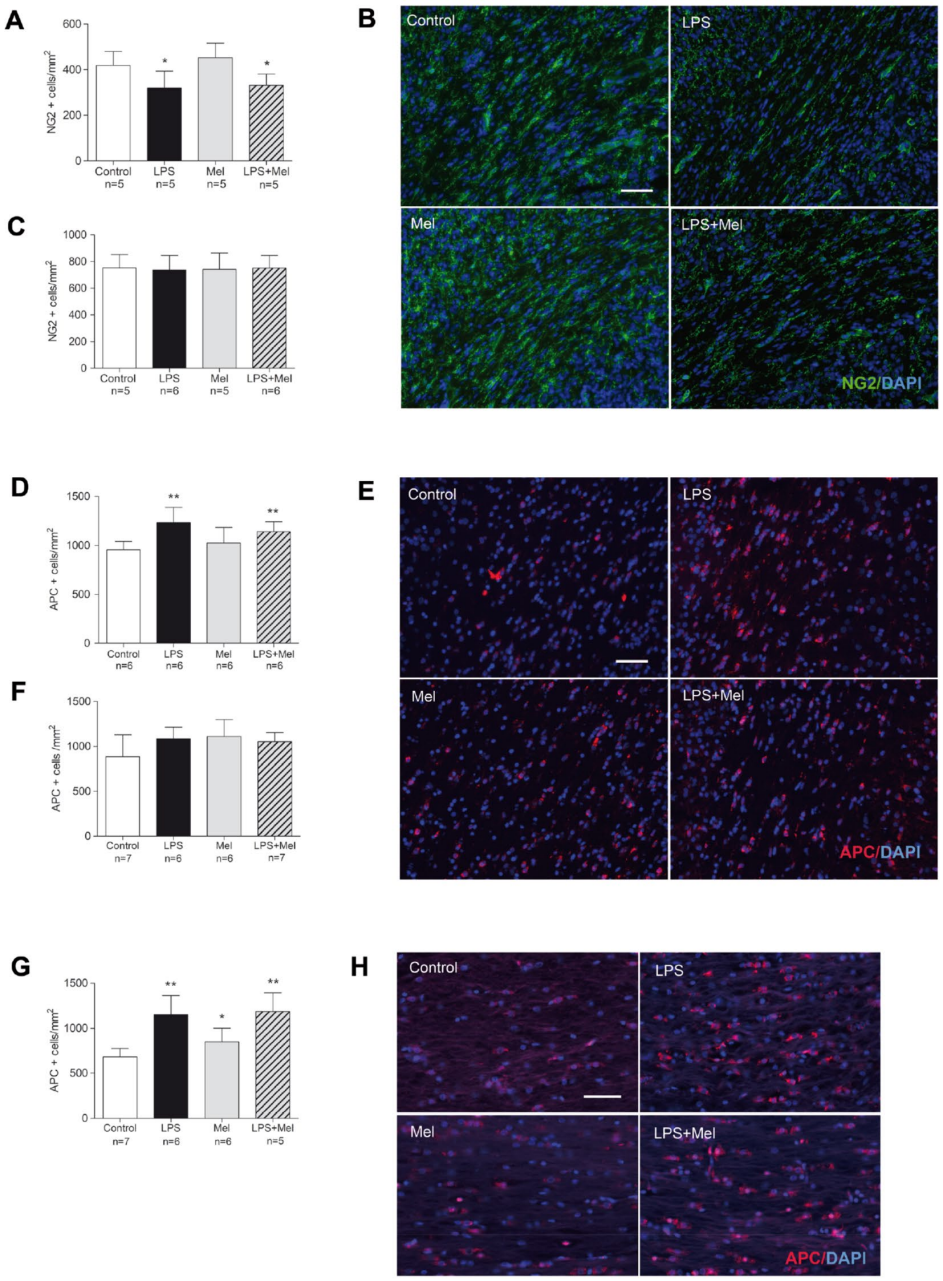
Melatonin failed to prevent the oligodendrocyte (OL) lineage alterations induced by the prenatal LPS challenge. Early OL populations stained by NG2 antibody were assessed within the external capsule in each experimental group on PND1 (**A**) and on PND7 brain sections (**C**), respectively. Photomicrographs of the NG2 immunofluorescence (green staining) coupled with DAPI counterstained (blue staining) within the external capsule at PND1 are reported in each experimental group (magnification 20, scale bar = 50 µm) (**B**). Mature OLs were stained by APC antibody (**E**,**H**). Photomicrographs of the APC immunostaining (red staining) coupled with DAPI counterstained (blue staining) within the external capsule at PND7 (magnification 20, scale bar = 50 µm) (**E**) and within the corpus callosum at PND21 (magnification 20, scale bar = 50 µm) (**H**) are reported. The APC-positive cells were counted within the external capsule of the PND7 (**D**) and PND21 (**F**) brains and within the corpus callosum at PND21 (**G**) in each experimental group. White, black, grey and hatched grey bars represented the Control, the liposaccharide portion of the *E. coli* membrane (LPS), the melatonin (Mel) and the LPS + Mel groups, respectively. Results are expressed as the mean of positive cells/mm^2^ ± standard deviation. Statistical analysis: One-Way-ANOVA test was performed associated with Bonferroni post-test, *p < 0.05 and **p < 0.01 in comparison with the Control group.

Significant reductions in the OPCs and the early Pre-OLs stained by NG2 antibody were observed within the external capsule in the brains of PND1 pups exposed to the LPS and to the LPS + Mel challenges (*p* = 0.03 and *p* = 0.04 in comparison with the Control group, respectively) (Fig. 4A,B). These decreases were transient as the NG2-positive cell populations were completely restored within the external capsule in the PND7 brains (Fig. 4C).

APC staining showed an increase in mature OLs within the external capsule of PND7 brains after the LPS challenge regardless of the melatonin exposure (LPS group: *p* = 0.008 and LPS + Mel group: *p* = 0.009 in comparison with the Control group) (Fig. 4D,E). This effect was transient and disappeared at PND21 within the external capsule (Fig. 4F). However, as was observed within the PND7 external capsule, an increase in the APC positive cells was also observed in the LPS and LPS + Mel groups within the corpus callosum at PND21 (LPS group: *p* = 0.002 and LPS + Mel group: *p* = 0.002 in comparison with the Control group,) (Fig. 4G,H). Interestingly, melatonin out of the inflammatory context (i.e., Mel group) promoted mature OLs within the corpus callosum in PND21 brains to a lesser extent than in the LPS and LPS + Mel groups (*p* = 0.03 in comparison with the Control group) (Fig. 4G,H).

### Melatonin did not effectively prevent LPS-induced hypomyelination in the PND7 brains

**Figure 5:**
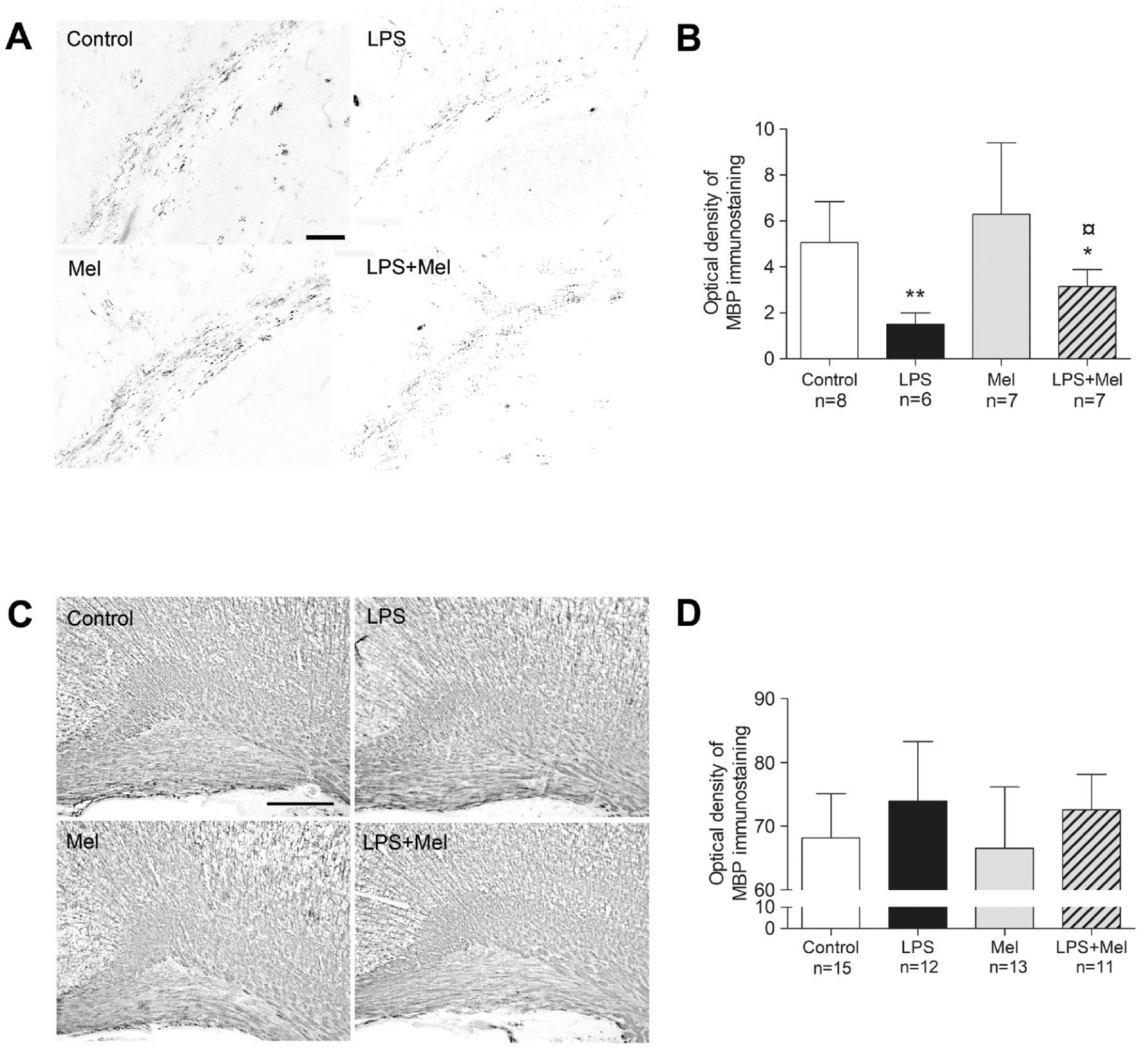
Melatonin did not effectively prevent LPS-induced hypomyelination within the external capsule at PND7. Myelin-basic protein (MBP) stainings were performed at PND7 (**A**,**B**) and at PND21 (**C**,**D**). The optical immunodensity of MBP staining was measured within the external capsule at PND7 (magnification 10, scale bar = 100 µm) (**A,B**) and within the cingulum at PND21 (magnification 10, scale bar = 200 µm) (**C**,**D**). White, black, grey and hatched grey bars represented the Control, the liposaccharide portion of the *E.coli* membrane (LPS), the melatonin (Mel) and the LPS + Mel groups, respectively. Results are expressed as the mean ± standard deviation. Statistical analysis: One-Way-ANOVA test was performed associated with Bonferroni post-test, *p < 0.05 and **p < 0.01 in comparison with the control group. ¤*p* < 0.05 in comparison with the LPS group.

A significant reduction in the MBP staining was observed within the external capsule in PND7 brains of the LPS group (*p* = 0.002 in comparison with the Control group) (Fig. 5A,B). Although the MBP staining was slightly more intense in the LPS + Mel group than in the LPS group (*p* = 0.03), melatonin failed to prevent LPS-induced hypomyelination within the external capsule at PND7 (*p* = 0.015 in comparison with the Control group) (Fig. 5A,B).

The intensity of MBP staining within the cingulum at PND21 was similar in each experimental group (Fig. 5C,D) and in other locations of the white matter, i.e., the external capsule, corpus callosum and sensorial-motor cortex (data not shown).

## Discussion

In this rat model mimicking chorioamnionitis, a melatonin treatment was given to the pregnant female in the acute phase of the inflammation/infection. The two target cells involved in the encephalopathy of the preterm infant, i.e., GABAergic interneurons and oligodendrocytes, were assessed through various brain regions and time-points in response to prenatal exposure to LPS and/or melatonin. Surprisingly, the preventive effect of melatonin in the inflammatory/infectious context varied according to cell type. Melatonin demonstrated a significant promoting action on GABAergic neurons in the neonatal rat brain in the inflammatory/infectious context, but failed to effectively prevent the impact of prenatal LPS on the OL lineage and the subsequent myelination. In the control population, although prenatal melatonin exposure did not lead to any specific effect on GABAergic neurons, melatonin slightly promoted mature OLs.

Our prenatal LPS challenge had strong impact on the pup survival and the pup weight at birth without any preventive effect of melatonin. The oxidative stress induced by LPS was suspected to be associated with these effects as they were prevented by N-acetyl cysteine, a glutathione precursor^20,21^. Intra-uterine fetal death and growth retardation were observed in pregnant mice exposed to LPS (75 µg/kg) at GD 15 and 17. Treatment of pregnant mice by melatonin either 5 or 10 mg/kg/dose just after and 3 h after LPS injections showed a dose–response reduction of fetal death. Conversely, this melatonin administration schedule failed to significantly restore fetal weight and to reduce the LPS-induced lipid peroxydation and glutathione depletion within placenta irrespective to the melatonin dose^22^. Melatonin as neuroprotective agent was usually administered to neonatal animals with dosing regimens from 10 to 20 mg/kg^2,4,7,8,23,24^. However, a significant and maximal promoting effect of melatonin on myelination was observed from the dose of 0.2 mg/kg in a model of unilateral uterine artery ligation in the pregnant rat at GD17^25^. Melatonin was daily administered from PND1 to PND3 with unit dose ranging from 0.002 to 20 mg/kg^25^. A feature of our model is that melatonin is injected to the pregnant rat during the late fetal life and not to the neonatal rat. Previous data demonstrated that melatonin freely crossed over placenta ^26^. Low-dose of melatonin, i.e., 0.1 mg/kg bolus plus 0,1 mg/kg/h over 6 h, administered to pregnant sheep experiencing umbilical cord occlusion showed a reduction of microglia activation and a restoration of immature OLs within periventricular white matter^27^. A fetal exposure to high flow of melatonin could be speculated in our model as pregnant rat were about 70 times heavier than PND1 pups. Our results showed that melatonin at the dose of 5 mg/kg/day in pregnant rats succeeded in preventing the pro-inflammatory response due to LPS at systemic and brain levels, in reducing cleaved-caspase-3 positive cells within the white matter and the LPS-induced sensitizing effect to a second excitotoxic brain insult in rat pups^19^. Therefore, these results supported this dosing regimen was sufficient to induce the melatonin neuroprotective effect in pups.

Prenatal LPS led to a significant reduction of GABAergic neurons within retro-spenial cortex at PND7. Previous results in our model showed an increase of apoptotic cell death within striatum in pups exposed to LPS but not within external capsule at PND1^18,19^. Then, these results suggested an excess of neuronal death early after LPS exposure that was prevented by melatonin. Apoptosis partly resulted from the release of cytochrome c from mitochondria to cytosol to activate the caspase cascade. Recent data demonstrated that melatonin exhibits a close relation with mitochondria to promote cell survival via receptor-dependent and receptor-independent pathways. Melatonin type 1 (MT1) receptors located at the outer membrane of mitochondria induced a G-protein-coupled-receptor inhibitory signal on the calcium-dependent release of cytochrome c ^28^. In parallel, melatonin directly induced sirtuin-1 (SIRT-1) pathway that upregulated Bcl2 protein, blocked Bax activity and down-regulated the cytochrome c release from mitochondria^29^. Previous data in our model showed that LPS inhibited SIRT-1 expression which was completely restore in LPS + Mel pups at PND1 supporting this hypothesis^19^. Furthermore, endogenous melatonin synthesis take place within the mitochondrial membrane of neurons suggesting an autocrine activation^28^. This endogenous synthesis of melatonin diminished with aging and made elderly patient more susceptible to infections or others inflammatory and neurodegenerative diseases^30,31^. Other life circumstances may modify this phenomenon like pregnancy^32^. Therefore, future research are needed to explore which innate and environmental factors influence this synthesis, the role of endogenous melatonin in the neonatal brain protection and the potential interaction with exogenous melatonin.

GABAergic neurons exhibited two patterns at PND21, depending on the brain regions. The GABAergic neuron population was higher in the LPS and the LPS + Melatonin groups within the retrospenial cortex and within the caudate-putamen whereas this increase was only observed in the LPS + Melatonin group within the dentate gyrus. Drury et al. reported a similar regional susceptibility of neurons to melatonin in fetal sheep exposed to a hypoxic-ischemic challenge^27^. This observation could be explained by the successive waves of GABAergic neuron production throughout brain development. Most GABAergic neurons are functional in the rat hippocampus during the late fetal period whereas the immature GABAergic interneurons are still migrating throughout the white matter to invade the cortical subplate and caudate nucleus^33–35^. These various responses could therefore indicate that the maturation stage of cells when insult occurs and when the neuroprotective agent is administered is a critical parameter to consider.

The prenatal LPS challenge induced an early decrease of OPCs followed by an increase of mature stages of OL lineage. This unbalance of OL lineage resulted in slowing down of the myelination process. This double-effect of LPS on OL lineage was previously described in others neonatal rodent models ^36,37^. In contrast to more mature OL stages, Pre-OLs are particularly vulnerable to oxidative stress due to glutathione deficiency and to the apoptotic effect of TNF-α^38,39^. After the initial phase of alleged Pre-OL death, an intense proliferative phase was described within the sub-ventricular zone ^37,40^. Neonatal LPS exposure also induced M2 polarization of microglia that was supposed to promote this proliferative effect ^37^. Then, microglia could play a central role in these early and late effects of LPS on OL lineage notably through cerebral pro-inflammatory cytokine expression ^40^. A pro-inflammatory response to LPS was observed in pups at PND1 in our model. Serum cytokine measurements in the serum of PND1 rat suggested that the prenatal LPS challenge induced a systemic pro-inflammatory response in pups. Furthermore, previous results in our model showed a significant increase of microglial cells double-stained with iba-1 and iNOS antibodies within the cingulum and a significant increase of IL-1β expression in pup brains at PND1 after LPS exposure^18,19^. Melatonin exhibited anti-inflammatory properties acting on various pathways. Melatonin interacted with microglia promoting its M2 polarization to the detriment of M1-subtype microglia^37,41^. Then, the late increase of cell population in brains of LPS + Mel pups similar to LPS pups could be linked to a joined action to favor M2-subtype microglia and cell proliferation. Previous data from our model showed that prenatal melatonin prevented the recruitment of activated M1-subtype microglia in the PND1 brains of rats exposed to LPS^19^. In parallel, the LPS activated through the Toll-Like-Receptor 4 (TLR4) pathway the NRLP3 inflammasome leading to strong pro-inflammatory cytokine expressions including IL-1β^41,42^. Melatonin interacted with this pathway to inhibit the NLRP3 inflammasome through SIRT-1 and the modulation of micro-RNA expressions ^41^. Previous published results in our model showed that the prenatal LPS challenge inhibited the SIRT-1 protein level in PND1 pup brain^19^. The association of melatonin to LPS restored SIRT-1 expression to the control group level^19^. Furthermore, melatonin showed a post-transcriptional regulation of miRNAs, i.e., miRNA-146a, miRNA-126, miRNA-34a^19^. Therefore, our prenatal melatonin administration schedule succeeded in neutralizing the pro-inflammatory effect of prenatal LPS on pup brains but failed to prevent OL lineage alterations. Our results suggest more complex interactions in vivo lightening the pivotal role of microglia with alternative pathways. In parallel, it is known that oligodendrocytes express melatonin receptors^25^. Although the direct action of melatonin on oligodendrocytes is not well known, melatonin is speculated to promote OL maturation^43^. This hypothesis fits with the specific effect of melatonin observed on mature OLs at PND21. Conversely to our data, melatonin previously demonstrated beneficial effects on OL lineage in another rat model using a LPS challenge. However, LPS and melatonin were injected to rat pups at PND5. In this context, melatonin succeeded in preventing the reduction of the Pre-OLs induced by LPS^8^. In parallel, melatonin succeeded in restoring normal myelination at PND14 in rat pups with intrauterine chronic hypoxia. Myelination was restored by a daily dose of melatonin from PND1 to PND3 by promoting OL maturation^25^. In contrast, previous studies showed that melatonin induced an incomplete restoration of immature OLs after a hypoxic-ischemic challenge in fetal sheep. As in our model, melatonin succeeded in reducing the microglial activation due to insult. This effect was observed both when melatonin was administered before or just after a hypoxic-ischemic insult and regardless of whether melatonin was infused to the pregnant female or to the fetus^27,44^. Therefore, the melatonin exposure in our model could be speculated to be too early considering the maturation of the OL lineage.

Although pre-clinical studies support melatonin use as neuroprotective agent in humans, clinical data in humans are scarce and focus on the term infant encephalopathy^45^. A recent study explored the kinetic of melatonin in preterm infants with the aim of supplementing them^46^. However, our experimental data pointed out that the neuroprotection due to melatonin is not constant in the immature brain.

Although our present data are mainly descriptive, the surprising lack of effectiveness of melatonin on oligodendrocytes in this context raises concerns and calls for further exploration.

## Conclusion

The effectiveness of melatonin in preventing the deleterious effect of an inflammatory/infectious challenge varied according to the cell lineage. The timing of exposure with respect to the maturation stages of cell lineages is likely to be critical to achieve more efficient neuroprotective effects of melatonin. Our results thus suggest that melatonin might not be an omnipotent neuroprotective drug. Moreover, our study showed that neuroprotection of the developing brain resulted from a complex machinery which might require more than a single-drug strategy. Finally, these data could suggest a modest neuroprotective effect of melatonin on extremely preterm infant brains.

## Animals and methods

### Animals and drugs

The time-pregnant Wistar rats (n = 70) used in this study were purchased from CERJ (Le Genest, France). They had free access to food and water and were bred at 22 °C with a normal light cycle.

**Figure 6:**
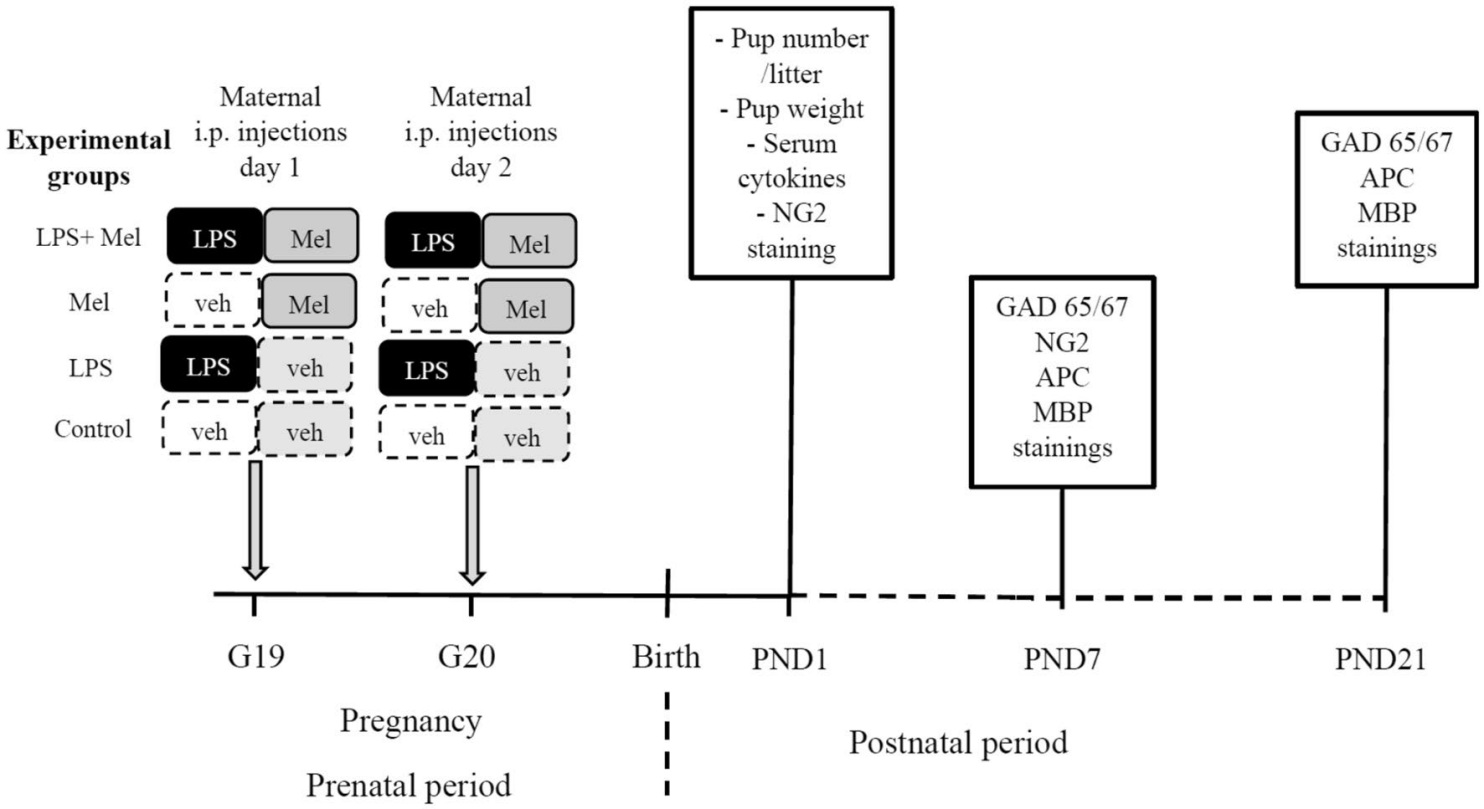
Experimental schedule. G: Gestational day, LPS: Liposaccharide portion of *E. coli* membrane, Mel: Melatonin, Veh: vehicle, i.p.: intraperitoneal, PND: Postnatal day.

The liposaccharide portion of Escherichia Coli membrane (LPS, *E. coli*, serotype 055:B5; Sigma Chemical Co., St. Louis, MO, USA) was diluted in saline solution (LPS vehicle) to a final concentration of 250 µg/ml. This LPS solution (300 µg/kg/dose) was then injected i.p. to pregnant rats at GD19 and at GD20. Melatonin (Sigma Chemical Co., St. Louis, MO, USA) was dissolved according to the manufacturer instructions and Drury P.P. et al*.* ^27^. A two-step dilution was performed to achieve the final concentration of melatonin of 1 mg/ml and 2% ethanol (melatonin vehicle). First, 50 mg of melatonin was diluted in 1 ml of 100% ethanol (50 mg/ml ethanol). Thereafter, 1 ml of this first solution was diluted in 49 ml of 0.9% sodium chloride. Melatonin (5 mg/kg/dose) was injected i.p. to pregnant rats at the same time as LPS injections (i.e., at GD19 and at GD20) in another abdominal site. Injections were performed at 10 a.m. The number of pregnant females treated in each experimental group was planned according to the number of pups required for each experiment to reach statistical threshold, i.e., seven pups per group for optical density and five pups per group for immunofluorescence experiments. As over mortality were previously observed in pregnant rats and fetuses treated by LPS, two times more female rats were treated with LPS and LPS plus melatonin^47,48^. Treatments were randomly assigned to female rats. The experimental groups were designed as follows: 1) The control group (Control) included pups from the pregnant rats treated by LPS and melatonin vehicles (n = 11); 2) The melatonin group (Mel) corresponded to pups from the pregnant rats treated by melatonin 5 mg/kg plus LPS vehicle twice (n = 10); 3) The LPS group (LPS) included pups from the pregnant rats treated by LPS 300 μg/kg plus melatonin vehicle twice (n = 23); 4) The LPS + Melatonin group (LPS + Mel) corresponded to pups from the pregnant rats treated by LPS 300 μg/kg plus melatonin 5 mg/kg twice (n = 26). Therefore, all rats were exposed to low doses of ethanol. The daily dose of LPS ranged from 110 to 125 µg. The daily dose of melatonin ranged from 1.8 to 2 mg. Male and female pups were considered for experiments with attempt to obtain sex ratio 1:1. Pups were counted in each litter at PND 1. Pup weights at PND 1 were also reported according to the maternal treatment. The experimental schedule is reported in Fig. 6.

### Serum cytokine measurements by immunoassay

Total blood were collected after decapitation of PND1 pups using glass capillaries. Blood samples were allowed to sediment overnight at 4 °C then centrifuged at 2000×*g*. Then, superficial layer corresponding to serum was carefully collected. Sera from pups of the same experimental group, litter and sex were mixed to obtain a minimal volume of 150 µl/ sample. Finally, samples were kept for long-term storage at − 80 °C until serum cytokine measurements. Measurements of IL1-β, IL-6, TNF-α and IL-10 levels in samples was performed using microbeads coupled with specific antibodies (Bio-Plex Pro Rat standard assay, Bio-Rad, Hercules, CA, USA). A cytokine-specific and quantitative fluorescent signal was produced after antigen fixation using a streptavidin-based reaction. The standard range was performed in duplicate and included nine decreasing points. Samples were diluted in three volumes of the sample diluent solution. Three wells were dedicated to each samples in the 96-well plate.

### Brain samples for immunochemistry experiments

Newborn rats were sacrificed by decapitation at PND 1 and 7. Brains were quickly removed from the skull and were post-fixed in 4% paraformaldehyde overnight at 4 °C. After 2 days in 10% sucrose—0.12 M phosphate buffer solution, the brains were embedded in a cooled 10% sucrose—7.5% gelatin solution before freezing. Finally, brains were cut coronally in 10 µm-thick sections.

Postnatal-day-21 rats were previously sedated in a chamber containing 2% isoflurane before decapitation. Brains were rapidly removed from the skull and immersed in a 4% formaldehyde solution for 4 days at room temperature. After dewatering in successive baths of 100% ethanol and xylen for 24 h, brains were embedded in paraffin and 10-µm thick coronal sections were performed.

### Immunochemistry protocol

The primary antibodies are reported in Table 1.

**Table 1:** Primary antibodies.

Protein targeted by primary antibody [cells or structure targeted]	Host and antibody type	Manufacturer/distributor (reference)	Concentration
Myelin-Basic-Protein (MBP) [myelin fibre]	Rat monoclonal	Merck-Millipore (MAB386)	1:500
Chondroitin Sulfate Proteoglycan (NG2) [Oligodendrocyte precursor cells and pre-oligodendrocytes]	Rabbit polyclonal	Merck-Millipore (AB5320)	1:200
Adenomatosis Polyposis Coli (APC, clone CC-1) [Mature oligodendrocytes]	Mouse monoclonal	Merck-Millipore (OP80)	1:2000
Glutamic Acid Decarboxylase 65 and 67 (GAD 65/67) [GABAergic neurons]	Rabbit polyclonal	Abcam (ab49832)	1:2000

For immunofluorescence experiments, the buffer for the antibody dilution contained 1X Phosphate-Buffer-Saline (PBS) solution with 1% donkey or goat serum depending on the secondary antibody plus 0.4% Triton X-100 to permeabilize the cell membrane except for MBP immunostaining. After 3 rinses in 1X PBS, antigen blocking was performed with 5% serum for 45 min. Then, the primary antibody was incubated overnight at room temperature. The second day, the appropriate secondary antibody was applied for 90 min (1:500 dilution for the Cy3 and Alexa 488 secondary antibodies). A counterstain by DAPI (1:10,000, Sigma-Aldrich, MO, USA) labeling the nucleus was performed at the end of the immunofluorescence protocol for NG2, APC and GAD 65/67 immunostainings.

For the MBP immunostaining on PND21 brain sections, deparaffination was performed followed by the antigen retrieval involving 40 min in citrate buffer at 93 °C. Antigen blocking was performed with 5% serum for 45 min. Then, the primary antibody was incubated overnight at room temperature. The second day, an Avidin–Biotin Complex supplied by the Vectastain ABC kit™ (Vector Laboratories, Burlingame, CA, USA) was incubated for 45 min. Then, diaminobenzidine (Sigma Chemical Co., St. Louis, MO, USA) was applied. The diaminobenzidine reaction was stopped as soon as the revelation degree was sufficient.

### Quantification of the MBP staining through optical density

The intensity of the myelin protein immunostaining was assessed by a densitometry analysis using the NIH ImageJ Software (1.46r version, NIH, USA). Optical density was deduced from grayscale standardized to the photomicrograph background. Four measurements / brain (2 in each hemisphere) were performed by blinded experimenters (GF, LS) in each brain region assessed.

### Count of the immunofluorescent cells

Cell counts were performed by blinded experimenters (GF, LS) within the brain structure of interest in duplicate (one measurement/ hemisphere) using the NIH ImageJ Software (1.46 r version, NIH, USA). Results are expressed as a number of positive cells/mm^2^.

### Statistical analysis

Quantitative data are expressed in mean ± standard deviation. A statistical comparison of our four experimental groups was performed through One-Way-ANOVAs with a subsequent Bonferroni post-test. The significance threshold was strictly under 5% (*p* < 0.05) for all analyses. The software program used for statistical analysis was GraphPad Prism (version 5.01 for Windows, GraphPad Software, San Diego, CA, USA).

### Ethics approval

All experimental procedures were carried out in compliance with the European Community Commission directive guidelines (86/609/EEC). The experimental protocol was approved by the Regional Ethics Committee (CEEA Val de Loire n° 19) under the reference n° 00022.01. This manuscript was written according to the ARRIVE guidelines^49^.

## References

[1] Wang Z (2018). Melatonin alleviates intracerebral hemorrhage-induced secondary brain injury in rats via suppressing apoptosis, inflammation, oxidative stress, DNA damage, and mitochondria injury. Transl. Stroke Res..

[2] Welin A-K (2007). Melatonin reduces inflammation and cell death in white matter in the mid-gestation fetal sheep following umbilical cord occlusion. Pediatr. Res..

[3] Balduini W (2012). The use of melatonin in hypoxic-ischemic brain damage: an experimental study. J. Matern. Fetal Neonatal Med..

[4] Hu Y (2017). Melatonin reduces hypoxic-ischaemic (HI) induced autophagy and apoptosis: An in vivo and in vitro investigation in experimental models of neonatal HI brain injury. Neurosci. Lett..

[5] Shi L (2018). Melatonin regulates apoptosis and autophagy via ROS-MST1 pathway in subarachnoid hemorrhage. Front. Mol. Neurosci..

[6] Ding K (2014). Melatonin stimulates antioxidant enzymes and reduces oxidative stress in experimental traumatic brain injury: The Nrf2–ARE signaling pathway as a potential mechanism. Free Radic. Biol. Med..

[7] Villapol S (2011). Melatonin promotes myelination by decreasing white matter inflammation after neonatal stroke. Pediatr. Res..

[8] Wong C-S, Jow G-M, Kaizaki A, Fan L-W, Tien L-T (2014). Melatonin ameliorates brain injury induced by systemic lipopolysaccharide in neonatal rats. Neuroscience.

[9] Robertson NJ (2013). Melatonin augments hypothermic neuroprotection in a perinatal asphyxia model. Brain.

[10] Jahnke G (1999). Maternal and developmental toxicity evaluation of melatonin administered orally to pregnant Sprague-Dawley rats. Toxicol. Sci..

[11] Andersen LPH (2016). Pharmacokinetics of high-dose intravenous melatonin in humans. J. Clin. Pharmacol..

[12] Colella M, Biran V, Baud O (2016). Melatonin and the newborn brain. Early Hum. Dev..

[13] Robertson NJ (2012). Which neuroprotective agents are ready for bench to bedside translation in the newborn infant?. J. Pediatr..

[14] Volpe JJ (2009). Brain injury in premature infants: A complex amalgam of destructive and developmental disturbances. Lancet Neurol..

[15] Robinson S, Li Q, DeChant A, Cohen ML (2006). Neonatal loss of γ-aminobutyric acid pathway expression after human perinatal brain injury. J. Neurosurg. Pediatr..

[16] Back SA (2001). Late oligodendrocyte progenitors coincide with the developmental window of vulnerability for human perinatal white matter injury. J. Neurosci..

[17] Favrais G (2011). Systemic inflammation disrupts the developmental program of white matter. Ann. Neurol..

[18] Rousset CI (2006). Maternal exposure to LPS induces hypomyelination in the internal capsule and programmed cell death in the deep gray matter in newborn rats. Pediatr. Res..

[19] Carloni S (2016). Melatonin modulates neonatal brain inflammation through endoplasmic reticulum stress, autophagy, and miR-34a/silent information regulator 1 pathway. J. Pineal Res..

[20] Buhimschi IA, Buhimschi CS, Weiner CP (2003). Protective effect of N-acetylcysteine against fetal death and preterm labor induced by maternal inflammation. Am. J. Obstet. Gynecol..

[21] Xu D-X (2005). Effect of N-acetylcysteine on lipopolysaccharide-induced intra-uterine fetal death and intra-uterine growth retardation in mice. Toxicol. Sci..

[22] Chen Y-H (2006). Melatonin protects against lipopolysaccharide-induced intra-uterine fetal death and growth retardation in mice. J. Pineal Res..

[23] Pang R (2021). Melatonin and/or erythropoietin combined with hypothermia in a piglet model of perinatal asphyxia. Brain Commun..

[24] Robertson NJ (2020). High-dose melatonin and ethanol excipient combined with therapeutic hypothermia in a newborn piglet asphyxia model. Sci. Rep..

[25] Olivier P (2009). Melatonin promotes oligodendroglial maturation of injured white matter in neonatal rats. PLoS ONE.

[26] Gomes PRL (2021). Maternal pineal melatonin in gestation and lactation physiology, and in fetal development and programming. Gen. Comp. Endocrinol..

[27] Drury PP (2014). Partial neural protection with prophylactic low-dose melatonin after asphyxia in preterm fetal sheep. J. Cereb. Blood Flow Metab..

[28] Suofu Y (2017). Dual role of mitochondria in producing melatonin and driving GPCR signaling to block cytochrome c release. Proc. Natl. Acad. Sci..

[29] Tarocco A (2019). Melatonin as a master regulator of cell death and inflammation: Molecular mechanisms and clinical implications for newborn care. Cell Death Dis..

[30] Tan D-X, Hardeland R (2020). Targeting host defense system and rescuing compromised mitochondria to increase tolerance against pathogens by melatonin may impact outcome of deadly virus infection pertinent to COVID-19. Molecules.

[31] Melhuish Beaupre LM, Brown GM, Gonçalves VF, Kennedy JL (2021). Melatonin’s neuroprotective role in mitochondria and its potential as a biomarker in aging, cognition and psychiatric disorders. Transl. Psychiatry.

[32] Ejaz H, Figaro JK, Woolner AMF, Thottakam BMV, Galley HF (2021). Maternal serum melatonin increases during pregnancy and falls immediately after delivery implicating the placenta as a major source of melatonin. Front. Endocrinol..

[33] Le Magueresse C, Monyer H (2013). GABAergic interneurons shape the functional maturation of the cortex. Neuron.

[34] Hennou S, Khalilov I, Diabira D, Ben-Ari Y, Gozlan H (2002). Early sequential formation of functional GABA(A) and glutamatergic synapses on CA1 interneurons of the rat foetal hippocampus. Eur. J. Neurosci..

[35] Xu G (2011). Late development of the GABAergic system in the human cerebral cortex and white matter. J. Neuropathol. Exp. Neurol..

[36] Xie D (2016). IL-1β induces hypomyelination in the periventricular white matter through inhibition of oligodendrocyte progenitor cell maturation via FYN/MEK/ERK signaling pathway in septic neonatal rats: IL-1β and periventricular white matter damage. Glia.

[37] Pang Y (2016). Early postnatal lipopolysaccharide exposure leads to enhanced neurogenesis and impaired communicative functions in rats. PLoS ONE.

[38] Pang Y, Cai Z, Rhodes PG (2005). Effect of tumor necrosis factor-? On developing optic nerve oligodendrocytes in culture. J. Neurosci. Res..

[39] Back SA, Gan X, Li Y, Rosenberg PA, Volpe JJ (1998). Maturation-dependent vulnerability of oligodendrocytes to oxidative stress-induced death caused by glutathione depletion. J. Neurosci..

[40] Pang Y (2010). Lipopolysaccharide-activated microglia induce death of oligodendrocyte progenitor cells and impede their development. Neuroscience.

[41] Hardeland R (2021). Melatonin and microglia. Int. J. Mol. Sci..

[42] Arioz BI (2019). Melatonin attenuates LPS-induced acute depressive-like behaviors and microglial NLRP3 inflammasome activation through the SIRT1/Nrf2 pathway. Front. Immunol..

[43] Long KLP, Breton JM, Barraza MK, Perloff OS, Kaufer D (2021). Hormonal regulation of oligodendrogenesis I: Effects across the Lifespan. Biomolecules.

[44] Yawno T (2017). The beneficial effects of melatonin administration following hypoxia-ischemia in preterm fetal sheep. Front. Cell. Neurosci..

[45] Ahmed J, Pullattayil SAK, Robertson NJ, More K (2021). Melatonin for neuroprotection in neonatal encephalopathy: A systematic review & meta-analysis of clinical trials. Eur. J. Paediatr. Neurol..

[46] Biran V (2019). Melatonin levels in preterm and term infants and their mothers. Int. J. Mol. Sci..

[47] Pujol Lopez Y (2015). Effects of subcutaneous LPS injection on gestational length and intrauterine and neonatal mortality in mice. NeuroImmunoModulation.

[48] Domínguez Rubio AP (2017). Maternal administration of melatonin exerts short- and long-term neuroprotective effects on the offspring from lipopolysaccharide-treated mice. J. Pineal Res..

[49] Kilkenny C, Browne WJ, Cuthill IC, Emerson M, Altman DG (2010). Improving bioscience research reporting: The ARRIVE guidelines for reporting animal research. PLoS Biol..

[50] Paxinos G, Watson CRR, Emson PC (1980). AChE-stained horizontal sections of the rat brain in stereotaxic coordinates. J. Neurosci. Methods.

